# Development of environment-friendly bio-based film with improved performance for food preservation using dialdehyde starch grafted konjac flour

**DOI:** 10.1016/j.fochx.2025.103121

**Published:** 2025-10-02

**Authors:** Xin Zhang, Jiawei Li, Hongyan Wang, Xiaojian Zhou, Liangjun Xiao, Hui Wang

**Affiliations:** a Southwest Forestry University, Yunnan Key Laboratory of Wood Adhesives and Glued Products, Kunming, Yunnan, PR China; b Zhejiang Academy of Forestry, Hangzhou, Zhejiang 310000, PR China; c Yunnan Academy of Forestry and Grassland, Kunming, Yunnan, 650201, PR China

**Keywords:** Konjac flour, Dialdehyde starch, Graft copolymerization, Structural features, Fresh-preservation application

## Abstract

Developing environment-friendly films by utilizing renewable bio-resource materials to substitute petroleum-based ones is regarded as an effective approach for addressing white pollution and its associated hazards. In this work, a redox system consisting of ascorbic acid/hydrogen peroxide (AA/H_2_O_2_) was established to promote the graft copolymerization of konjac flour (KF) and dialdehyde starch (DAS) for the preparation of bio-based films. The results indicated that the overall performance of the film was significantly improved after grafting DAS onto KF. Specifically, when the DAS content was 25 % of the KF weight, the film presented the optimal properties: a moisture content of 10.2 %, a water contact angle of 87.17°, a tensile strength of 33.08 MPa and a biodegradation rate of 93.16 %. Additionally, the weight loss of banana in a 7-day experiment was only 15.67 %, taking the film as a preservative material. Meanwhile, the film's dense and uniform structure also enhances its thermal properties.

## Highlights

• A series of bio-based films were fabricated using konjac flour (KF) grafted dialdehyde starch (DAS) polymer as raw material.
• In this work, the prepared films possessed excellent performance, especially thermal stability, tensile strength, biodegradability and preservation property.
• By adjusting content of DAS, the film with 25 % DAS content exhibited the optimal performance.

## 1 Introduction

Humans rely heavily on food for survival, and a reasonable and effective food packaging system plays a crucial role in preventing food spoilage and extending their shelf life (Xie et al., 2024). Advances in synthetic plastics and film technology have dramatically simplified food packaging and preservation (Hernández et al., 2024). However, their non-degradability and low reuse rate have triggered critical environmental pollution and human health concerns (Cheng et al., 2024; Zhang, Jiang, et al., 2024). Hence, to develop environmentally friendly packaging materials is considered as an effective way to address these issues (Latif et al., 2024). As a result, an increasing number of research efforts have focused on exploring bio-based films, and numerous studies on replacing petroleum-based materials with natural bio-resources for developing food packaging materials are rapidly expending (Farhan & Hani, 2017).

Polysaccharide, which are widely distributed in plant roots, stems, and fruits, represent the most abundant polymers in nature (Li et al., 2021). Owing to their outstanding characteristics such as biodegradability, biocompatibility, non-toxicity, and antioxidant activity, they have found extensive application in various industries, including food, agriculture, water treatment, bioengineering medicine and textiles (Żołek-Tryznowska et al., 2024). Among them, konjac glucomannan (KGM) has been employed as a principal ingredient for manufacturing food packaging films due to its remarkable film-forming properties (Huang et al., 2016). It is well known that konjac tubers contain not only the non-ionic polysaccharide konjac glucan but also other components such as starch, cellulose and proteins, all of which can also be used in the production of bio-based films (Zhang, Li, et al., 2024). Therefore, an idea of using konjac raw flour (KF) directly as a film-forming material has been proposed. If successful, the process of extracting KGM could be avoided, implying that all energy consumption during extraction would be saved. Furthermore, the utilization efficiency of KF resources could also be greatly improved. Compared to KGM as a film-forming raw material, the cellulose and protein components contained in konjac raw flour could help enhance the mechanical properties and water resistance of the film(Tatirat & Charoenrein, 2011). In light of these considerations, KF was chosen as the primary film-forming substance for the fabrication of bio-based films in the present study.

Actually, the development of high-quality bio-based films using only biomass raw materials remains a challenge, as bio-materials typically exhibit deficiencies in water resistance and possess relatively low strength (Lin et al., 2024). Therefore, implementing physical or chemical modifications is regarded as the primary approach to enhance the overall performance of bio-based films (Yao et al., 2024). For KF, aldehydes compounds such as formaldehyde, glutaraldehyde, etc., have been confirmed as effective modifiers for improving the overall performance of films (Easdani et al., 2024). However, these aldehyde compounds are also unsuitable as modifiers for food packaging films because they are not safe to the human. Nevertheless, inspired by this, in order to harness the cross-linking capabilities of aldehyde group while maintaining a more environmentally friendly and safe system, the employment of dialdehyde starch (DAS) as a modifier represents an ideal alternative. This is because DAS not only contains a significant quantity of active aldehyde groups that are non-toxic but has also been applied extensively in food packaging, drug transport, wood adhesives, thermoplastic manufacturing, heavy metal ion adsorption and enzyme immobilization (Miao et al., 2023). However, the improvement is restricted due to limited extent of cross-linking. So, to address this limitation, increasing the degree of chemical cross-linking among diverse biomass raw materials as much as possible has emerged as a primary method for augmenting the performance of bio-based films in recent years. Among available strategies, the grafting method of different raw materials is relatively straightforward, cost-effective, and environmentally benign, thus gaining wide acceptance (Du et al., 2023). Based on the reported literature, the grafting methods could be categorized into free radical-induced grafting, enzyme grafting and carbodiimide-based coupling grafts (Mohamady Hussein et al., 2023). Among these, the free radical grafting approach within the AA/H_2_O_2_ redox system is regarded as a promising strategy (Liu et al., 2018), as this system could rapidly promote the grafting process, react completely without generating pollutants and hazardous substances, and enhance the biodegradability of the resulting film. Consequently, the AA/H_2_O_2_ redox system was employed to achieve extensive chemical cross-linking between DAS and KF, with the aim of fabricating high-performance, environmentally friendly, bio-based films for food packaging.

Furthermore, the prepared film using grafted DAS onto KF as the film-forming substrate was expected to exhibit enhanced performance, particularly in mechanical properties and water resistance. These characteristics were the essential metrics for evaluating bio-based films. Notably, compared with our previous work (Zhang et al., 2024), the performance of films showed significant improvement. Hence, an excellent and environmentally friendly bio-based film could be developed using the AA/H_2_O_2_ redox system compared to blending method, which was very important advancement for food preservation application. For this, to achieve optimal performance, a series of bio-based films were prepared with varying the DAS content. And their physical and mechanical properties were investigated, the structural features were analyzed using fourier transform infrared spectroscopy (FT-IR), X-ray diffraction (XRD), and scanning electron microscopy (SEM). Considering their potential application as fruit preservation films, the preservation effect and degradability were also evaluated.

This work aims to provide an effective method for developing high-performance bio-based films for food preservation, using KF as the raw material by grafting DAS via a green AA/H_2_O_2_ redox system. It also seeks to contribute to improving the utilization efficiency of KF.

## 2 Materials and methods

### 2.1 Materials

Konjac flour (KF, food grade) was purchased from Yunnan Lushan Biological Co., Ltd. It had a weight-average molecular weight of 20,000 g/mol and a solubility of 95 %; Double aldehyde starch (DAS, analytical grade) in white powder form was supplied by Dongguan Xin'an New Chemical Materials Co., Ltd. Its weight-average molecular weight was 16,547 g/mol with a straight-chain starch of 34.8 %, moisture content ≤17 %, whiteness of 80 %, pasting temperature of 62 °C ∼ 70 °C, and pH of 6.5–7.5. The 30 % hydrogen peroxide (H_2_O_2_) solution was supplied by Jinshan Chemical Co. (analytical grade). Ascorbic acid (AA, analytical grade), glycerol, anhydrous sodium carbonate (analytical grade), and anhydrous ethanol (analytical grade) were all obtained from Guangdong Guanghua Technology Co. All reagents except KF were of analytical grade and were utilized without any further purification. And the distilled water used in this work was self-prepared in our laboratory.

### 2.2 Preparation of the bio-based films

#### 2.2.1 Grafting between KF and DAS

At room temperature, 10 g of KF was dissolved in a 15 % alcohol solution under magnetic stirring for 20 min. Then, 6.6 wt% AA and 150 wt% H_2_O_2_ based on KF weight were added, and the mixture was stirred for another 30 min. Thereafter, varying amounts of DAS were incorporated into the mixture, which was continuously stirred for 24 h at 25 °C. Subsequently, the pH was adjusted to 7 using a 20 % Na_2_CO_3_ solution, followed by an additional 20 min of stirring. Finally, the liquid slurry was washed twice with anhydrous ethanol and then freeze-dried at −50 °C for 24 h. The obtained polymers were named as DAS0-g-KF, DAS5-g-KF, DAS15-g-KF, DAS25-g-KF, DAS35-g-KF, and DAS45-g-KF, corresponding to the weight ratios of DAS to KF (0 %, 5 %, 15 %, 25 %, 35 %, and 45 %). And all samples were stored in sealed containers for later utilization.

#### 2.2.2 Films preparation

**Scheme 1:**
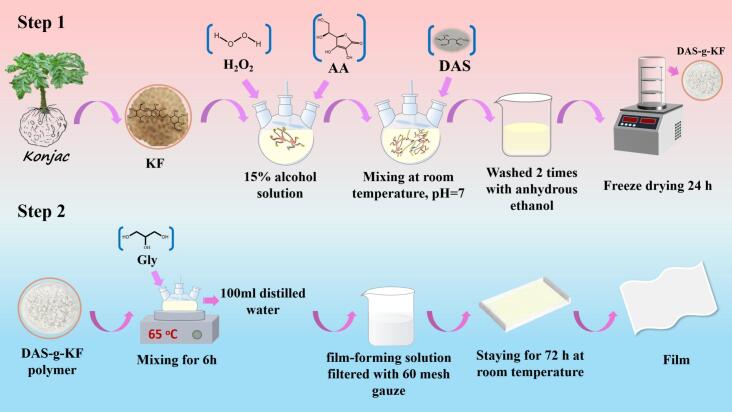
Preparation process of bio-based films.

2 g of DAS-g-KF polymer were precisely weighed and dissolved in 100 mL of distilled water, followed by the addition of 0.3 g of glycerol. The resulting solution was heated to 65 °C under continuous stirring in a water bath and maintained at this temperature for 6 h. The solution was then filtered through 60-mesh gauze and poured into a 15 × 15 cm polytetrafluoroethylene (PTFE) mold. After being air-dried at room temperature for 72 h, the bio-based films were carefully peeled off from the mold. Accordingly, the prepared films were labeled as DAS0-g-KF, DAS5-g-KF, DAS15-g-KF, DAS25-g-KF, DAS35-g-KF, and DAS45-g-KF corresponding to the type of polymer used. All these films were conditioned at 20 °C and 50 % relative humidity for 24 h prior to testing. The preparation process of the bio-based films is illustrated in Scheme 1.

### 2.3 Characterization

#### 2.3.1 Thickness and moisture content

Modified from the method described by Liu (Liu et al., 2023), 5 cm × 5 cm film sample was randomly selected and measured using an Everett digital micrometer (Model BC801, Henan Bangtong Special Gauge Co., Ltd.). The mean value of five measurements was designated as the ultimate measurement outcome for each specimen.

The moisture content (MC) of the film was modified and determined according to Ton-That (Ton-That et al., 2025). The initial mass of a 2 cm × 2 cm film sample was recorded as m_0_ (g), followed by drying the sample in an oven at (103 ± 2)°C until the mass keeping consistent, and the mass was recorded as m_1_ (g). Each sample was tested three times, and the average value was calculated using Formula (1) for data analysis.

(1) $$\mathrm{MC}=\frac{m_{0}-m_{1}}{m_{0}}\times 100\%$$

#### 2.3.2 Opacity testing (OP)

According to the method proposed by Wang (Wang et al., 2023), an ultraviolet spectrophotometer (WFZUV-4802S) was used to measure the opacity (OP) of the film at 600 nm. 4 cm × 1 cm film sample was placed in an ultraviolet spectrophotometer cell and tested at least three times. The opacity (OP) was calculated using formula (2). The final result was from the average value of three tests per sample.

(2) $$\mathrm{OP}=\frac{A}{x}$$

where A was the absorbance value at wavelength 600 nm(Abs); *x* was the thickness of the film (mm).

#### 2.3.3 Water vapor permeability (WVP)

The water vapor permeability (WVP) of the film was determined according to the method proposed by Xu (Xu et al., 2024). A total of 3 g of anhydrous calcium chloride was placed into the test cup, then the cup opening was sealed with a circular film sample with an area of 234.94 mm^2^, and stored in a desiccator containing 1 L of distilled water at a temperature of 23 ± 0.2 °C. After 24 h, the test cup was precisely weighed, and the increase in water mass was recorded as W. Calculations was performed using formula (3), with each sample tested three times, and the average value was used for data analysis.

(3) $$\mathrm{WVP}=\frac{W\times x}{A\times ∆p\times t}$$

where W was the weight change, g; x was the film thickness, mm; A was the area of film specimen, mm^2^; ∆p was a water vapor pressure Pa; t was the time, s.

#### 2.3.4 The oxygen permeability

The oxygen permeability (OP) of the film was determined according to the method proposed by Xu (Xu et al., 2024). A 3 g sample of deoxidizing agent powder, with a mass ratio of reduced iron powder, sodium chloride, and activated carbon of 0.5:1.5:1, was placed in a test bottle and sealed with the film sample. The initial weight was recorded as M_1_ (g). The bottle was then placed in a desiccator maintained at 90 % relative humidity and a temperature of 25 ± 0.2 °C for 48 h, after which the weight was recorded as M_2_ (g). The OP of film was calculated according to formula (4). Each sample was tested three times, and the average value was taken as the final result.

(4) $$\mathrm{OP}=\frac{M_{2}-M_{1}}{t\times s}$$

where: M_1_ is the initial weight of the bottle, g; M_2_ is the weight of the bottle after 48 h, g; t is the placed time, s; s is the film surface area, mm^2^.

#### 2.3.5 Water solubility (WS)

The determination of water solubility (WS) of the film was based on the modified method proposed by Xie (Xie et al., 2021). The film sample (2 cm × 2 cm) was first dried in an oven at 105 °C until a constant weight (m_1_) was achieved. Then, the film was immersed into 30 mL distilled water, and maintained at room temperature for 24 h. The insoluble components was filtered out and dried again at 105 °C until reaching a constant weight (m_2_). Based on the formula (5), WS was calculated at least three times for each sample, and its average value for a final analysis.

(5) $$\mathrm{WS}=\frac{m_{1}-m_{2}}{m_{1}}\times 100\%$$

#### 2.3.6 The water contact angle (WCA)

The water contact angle (WCA) of the film was measured using a contact angle meter (Dat physical DCAT21) according to Zhang's method to evaluate its hydrophobicity (Zhang, Wang, et al., 2024). The size of tested film was 5 cm × 1 cm, and distilled water was used as the testing reagent. Each sample was tested three times, and the average value was taken for data analysis.

#### 2.3.7 Mechanical properties testing

According to the testing method of the mechanical testing machine, 15 mm × 20 mm film strips were measured at a speed of 50 mm/min, at room temperature (25 °C) and a relative humidity of 45 %–55 % on a mechanical testing machine (SUST 5569). And the tensile strength (TS), the elastic modulus (EM) and elongation at break (EAB) of films could be obtained. Each type of film was tested five times in parallel, and their average value was used as the test result.

#### 2.3.8 Fourier transform infrared spectroscopy (FT-IR)

The structural characteristics of film was performed using fourier transform infrared spectroscopy-attenuated total reflection (ATR) technology (Thermo Scientific Nicolet IS50，China). Each sample was scanned in the range of 500–4000 cm^−1^, at 32 scans and a resolution of 4 cm^−1^. The final spectrum was normalized before further analysis.

#### 2.3.9 X-ray diffraction (XRD)

X-ray diffraction spectra of the thin films were measured using an Ultima IV X-ray diffractometer (XRD-7000, Hitachi, Tokyo, Japan), equipped with Cu-Kα radiation, at a scanning rate of 2°/min and a scanning angle range between 5° and 90°.

#### 2.3.10 Scanning electron microscopy (SEM)

The microstructure of the film was determined using a scanning electron microscope (SEM, Zeiss Sigma 300, Carl Zeiss, Germany). The film(1 cm × 4 cm) was subjected to low-temperature fracture treatment using liquid nitrogen, and the fracture surface was covered with gold. The scanning process was performed at an acceleration voltage of 20 kV.

#### 2.3.11 Thermal property testing of the films

The thermal property of film was tested on a thermogravimetric analyzer (TG 209F1, NETZSCH, Germany). 5–10 mg film sample was weighed and heated within a temperature range from 30 °C to 800 °C at a heating rate of 10 °C/min under a nitrogen atmosphere.

#### 2.3.12 Biodegradability testing of the films

The biodegradability of the film was determined but a few small modifications were made according to the burial method as described in the literature (Deng et al., 2023). Specifically, the soil was collected from an outdoor site located at 102° longitude and 25° latitude, with a pH value of 6.25 and a moisture content of 32 %. To ensure controllable experimental conditions, the degradation experiment was conducted indoors at a temperature maintained between 22 and 25 °C. The film was cut into pieces measuring 50 mm × 100 mm, dried to a constant weight (M0, g), placed in plastic mesh bags, and buried 150–200 mm below the soil surface. Every 5 days, the samples were carefully removed from the soil. The soil residues adhering to the film surface were removed by rinsing with distilled water, followed by drying the samples in a constant-temperature oven at 50 °C for 6 h. The dried samples after biodegradation were labeled as M_1_ (g). The biodegradation rate of the film was calculated using formula (6).

(6) $$\text{Biodegradation rate}=\frac{M_{0}-M_{1}}{M_{0}}\times 100\%$$

#### 2.3.13 Application performance of the films

The application performance of the film was investigated by evaluating the freshness-preserving effects on bananas, and the weight loss of the bananas was assessed according to Zhao (Zhao et al., 2022). Fresh bananas with similar size, uniform ripeness, and no mechanical damage were randomly divided into seven groups. One group served as the blank control group without any treatment, while the remaining each group was wrapped in film and stored at room temperature for seven days. Daily photographs of the bananas were taken using a Huawei Mate 60 smartphone, and their weights were recorded. The banana weight loss rate was measured using formula (7).

(7) $$\text{Weight loss}=\frac{W_{1}-W_{2}}{W_{1}}\times 100\%$$

where W_1_ is the weight of the strawberries before packing, and W_2_ is the weight of the strawberries after elapse of a specific time after packing.

#### 2.3.14 Statistical analysis

All experimental measurements were taken from three to five samples test, and statistical analysis was performed by Origin 2018 (Northampton MA), processing data using analysis of variance.

## 3 Results and discussion

### 3.1 Physical properties

**Fig. 1:**
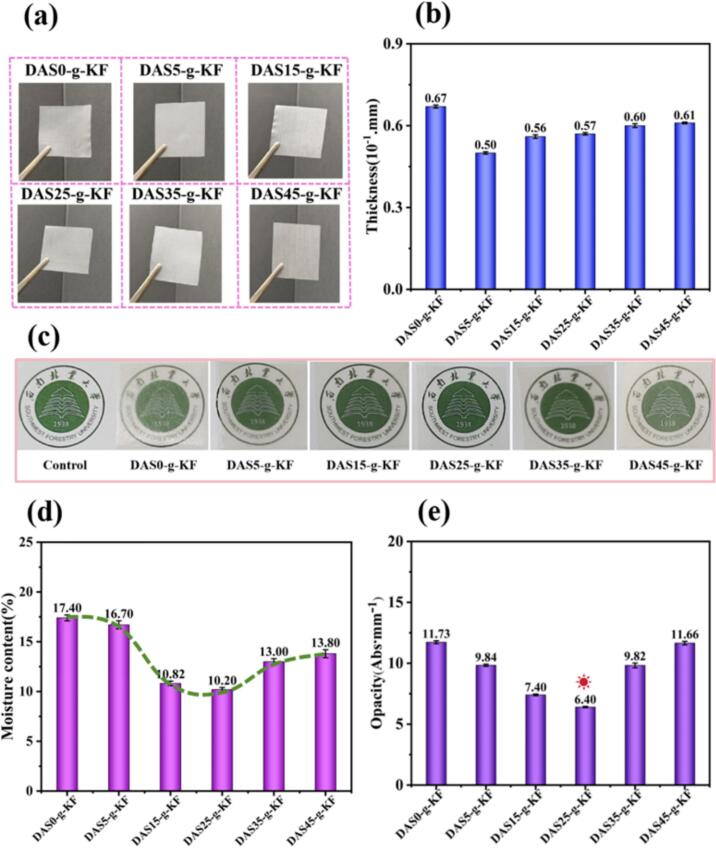
The physical properties of the films on appearance (a); thickness (b); transparency comparison (c); moisture content (d), and opacity (e).

A series of bio-based films were fabricated with varying amounts of DAS following the process illustrated in Scheme 1. The physical properties of these films, including appearance, thickness, transparency, moisture content, and opacity, were investigated, and the obtained results were presented in Fig. 1. As shown in Fig. 1a, all films were similar in appearance and were colorless. However, the DAS content significantly affected the thickness, moisture content, and opacity of the films. As indicated in Fig. 1b, the thickness of the films initially decreased and then increased with increasing DAS content, suggesting that DAS was a major factor influencing film thickness. The initial decrease could be attributed to a reduction in the spatial distance between molecules due to increased intermolecular packing density of the film after grafting DAS onto KF. In contrast, higher amounts of unreacted DAS led to an increase in film thickness.

In combination with the changes in film transparency shown in Fig. 1c, it could be observed that the amount of DAS had a remarkable impact on the transparency of the films under uniform conditions. The DAS25-g-KF film exhibited the highest optimal transparency, as the picture appeared sharper. These results implied that the difference in the internal structures of the films led to variations in light absorption and reflection capacities. Furthermore, the DAS25-g-KF film also had the lowest of the moisture content and opacity as the results in Fig. 1d and e. This indicated that an ideal cross-linking system could be achieved in the film when 25 % DAS was grafted with KF, as chemical cross-linking could reduce free hydrophilic groups, optimize the structure, and improve the stability of the film in humid environments (Ton-That et al., 2025). Moreover, the lower film opacity would provide application possibilities for food, vegetable and fruit packaging.

### 3.2 Water resistance analysis

**Fig. 2:**
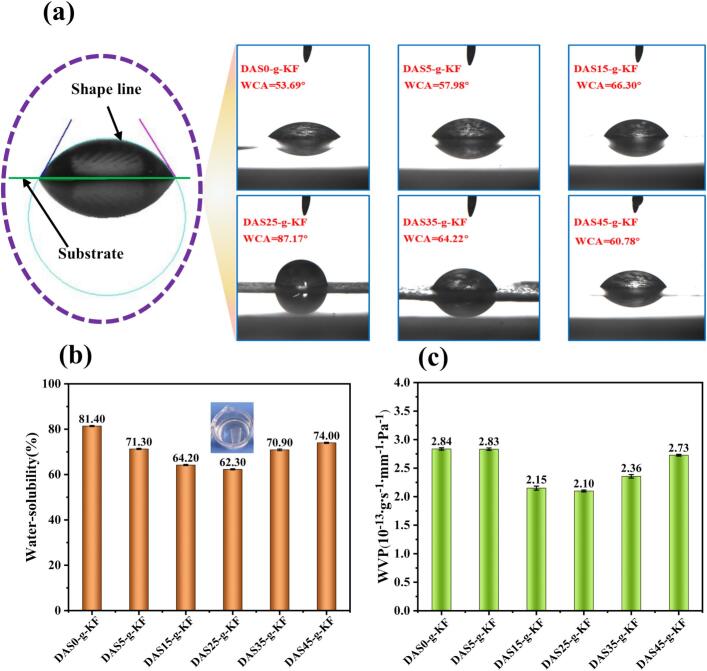
Final values of the films of WCA (a); WS (b); and WVP (c).

To further evaluate the stability of these films under humid conditions and their water resistance performance, the surface wettability, water solubility, and water vapor transmission rate were determined, and all the results were presented in Fig. 2. The hydrophobicity of the film surface is a key factor in preventing undesirable changes caused by an increase in the moisture content of the protected food (Balasubramaniam et al., 2020). Therefore, the surface wettability of the films was tested using the static contact angle measurement method with water serving as the solvent, and the results were shown in Fig. 2a. Overall, the surface of the films exhibited a certain degree of hydrophilicity, which was greatly influenced by the DAS content. Similar to the trend observed in physical properties, the water contact angle (WCA) of all films increased to varying degrees, with the DAS25-g-KF film showing the largest WCA value (87.17°). Compared to the previous prepared film by mixing KF with DAS (Zhang, Jiang, et al., 2024), the hydrophobicity of the films produced by the method presented in this work, had been significantly improved. Thus, the stated method in this work was more effective for developing high-performance KF-based films.

Additionally, the water solubility and water vapor permeability (WVP) of the films were determined as well. As shown in Fig. 2b, the water solubility of the films improved significantly compared to the film without DAS addition, particularly the DAS25-g-KF film exhibited the best performance. This indicated that both the DAS content and its cross-linking degree positively influenced the water solubility of the film. It could be reasonably inferred that an optimal chemical cross-linking structure could be formed between DAS and KF when DAS content was 25 %. However, by comparing with petroleum-based film, there remains considerable potential for improvement in the water solubility of these bio-based films. Completely eliminating hydrogen bond interactions between free water and biomass materials remained a significant challenge for bio-based film (Yang et al., 2025). Furthermore, the WVP results of the films in Fig. 2c followed a trend highly consistent with the water solubility, and the DAS25-g-KF film showed the lowest WVP value. In general, a denser internal structure tended to hinder penetration under identical conditions (Zhang, Li, et al., 2024). Therefore, it could be concluded that the films with a lower WVP value possessed a compact structure and a high degree of cross-linking.

### 3.3 Mechanical properties

**Fig. 3:**
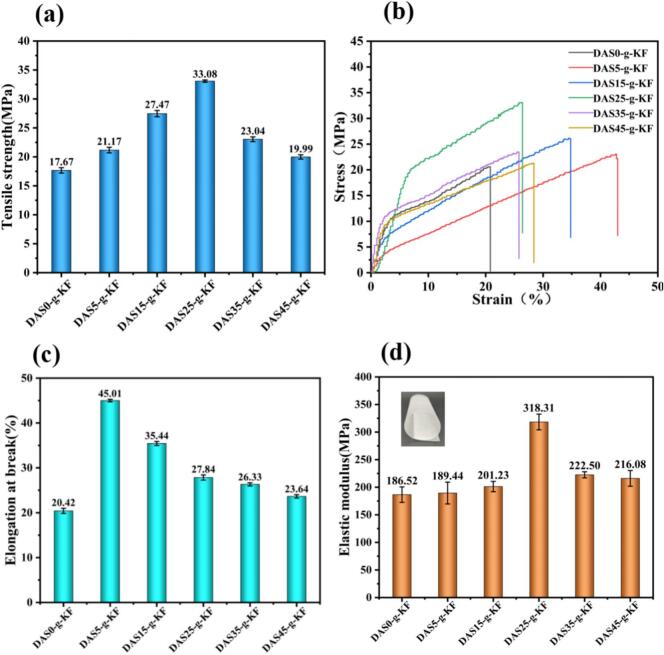
The mechanical results of the films including tensile strength (a); strain-stress curves (b); elongation at break (c); elastic modulus (d).

Mechanical properties played a pivotal role in the practical application of films. The mechanical test results of bio-based films, with the DAS-grafted KF polymer as the main film-forming substance, were represented in Fig. 3. Evidently, the overall mechanical properties of the films, including tensile strength (TS), elastic modulus (EM), and elongation at break (EAB), were enhanced with the introduction of DAS. As the DAS content increased, the EAB of the films gradually decreased, indicating that DAS would impart rigidity to the films, likely due to its macromolecular structure. Moreover, enhanced deformation resistance could be observed in Fig. 3b. Specifically, when the DAS grafting content was at 25 %, both TS and EM of the film reached the optimal values: 33.08 MPa and 318.31 MPa, with an percentage increases of 70.66 % and 87.21 %, respectively, compared to the DAS0-g-KF films. However, when the DAS content exceeded 25 %, the increase of TS and EM would decline progressively. All these changes indicated that DAS grafted KF enhanced intermolecular chemical cross-linking under appropriate conditions, achieving an optimal balance in intermolecular interactions and resulting in superior properties. Beyond this point, excessive DAS content increased the stiffness of the films, which was primarily attributed to the altered equilibrium between chemical cross-linking and the macromolecular interactions within the film.

In contrast to some results reported in the literature, although the EAB of the films in this study was relatively low, the TS values were higher than those of most bio-based composite films (Sun et al., 2025). This implied that the grafted copolymerization between KF and DAS may restrict the mobility of the molecular chains while enhancing the rigidity at the expense of some plasticity. Nevertheless, the overall flexibility of the films remained suitable for packaging applications.

### 3.4 The structural analysis

**Fig. 4:**
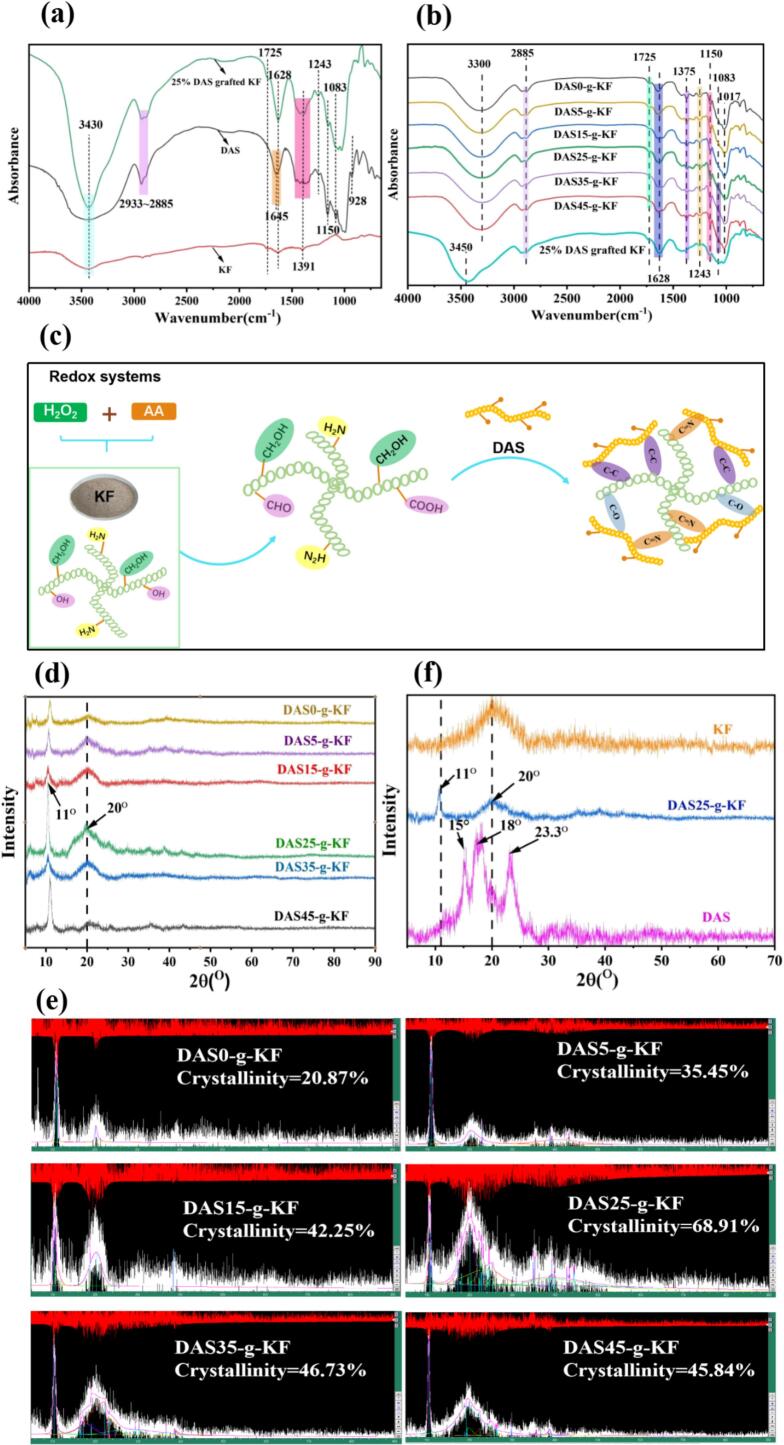
FT-IR spectra of KF, DAS and 25 % grafted KF (a); FT-IR spectra of films with different DAS content, and 25 % grafted KF (b); Schematic diagram between KF and DAS under redox systems (c); The XRD results of prepared films (d); The crystallinity value of films with different DAS content (e); The XRD curves of KF, DAS and DAS25-g-KF (f).

Based on the physical and mechanical results of the films, the prepared bio-based films exhibited a relatively dense structure, and the chemical cross-linking was likely formed between DAS and KF. To further explore their structural characteristics and gain a deeper insight into the film formation mechanism, FT-IR, XRD, and SEM were employed to detect the structural features of the films, and the results were shown in Fig. 4. The peaks in the FT-IR spectra were assigned according to the characteristics of the raw materials used and relevant literature. The FT-IR spectra of KF, DAS, and their grafted polymers were shown in Fig. 4a and b. As shown in Fig. 4a, the broad peak around 3430 cm^−1^ corresponded to -O-H and -N-H stretching vibrations (Yang et al., 2024). A strong peak could be observed in the 25 % DAS grafted KF polymer. The peaks in the range of 2933–2885 cm^−1^ were typically attributed to C—H stretching vibrations in -CH_2_- or -CH- groups (Shah Bukhary et al., 2024). The peaks at 1645 cm^−1^ and 1628 cm^−1^ were associated with -N-H bending and C=N stretching vibrations (Wang et al., 2025), while the peak at 1391 cm^−1^ belonged to -C-N and -C-O vibrations (Wang et al., 2022). And the peaks at 1243 cm^−1^, 1150 cm^−1^, 1083 cm^−1^ and 928 cm^−1^ were commonly assigned to C—O deformation vibrations (Marques et al., 2024). It could be observed that the peak intensity in the 2933–2885 cm^−1^ region was weaker in the 25 % DAS grafted KF polymer compared to pure DAS. Similarly, the peaks at 1150 cm^−1^ and 1083 cm^−1^ also exhibited reduced intensity in the grafted polymer. However, the peaks at 1628 cm^−1^ and 1243 cm^−1^ were stronger in the 25 % DAS grafted KF polymer than in DAS alone. These spectral changes suggested the formation of strong intermolecular hydrogen bonds within the DAS grafted KF polymer, as well as possible oxidation, Schiff base and aldol condensation reactions between aldehyde group (-CHO) and hydroxymethyl group (-CH_2_OH) in DAS and amino groups (−NH_2_) in KF, leading to the formation of macromolecular polymers. And a possible binding modes were illustrated in Fig. 4(c).

Furthermore, Fig. 4b presented the FT-IR spectra of films with varying DAS contents. It could be observed that the characteristic peak of the grafted polymer at 3430 cm^−1^ shifted to a lower wavenumber of 3300 cm^−1^, and the peak became broader and weaker, indicating that the -OH or -NH_2_ groups underwent further cross-linking during preparation of film-forming solution and film-forming process with the help of glycerol. This phenomenon indicated that the presence of strong hydrogen-bonding interactions in the final films, which may be one of factors restricting the improvement of water resistance. Moreover, a significant weakening was noted at the peak of 1628 cm^−1^ after film formation, suggesting that intramolecular cross-linking were still conducted during the film-forming process. Meanwhile, the peak at 1725 cm^−1^ gradually disappeared and the peak in the range of 1645–1628 cm^−1^ progressively broadened, indicating the cross-linking between KF and DAS persisted.

To further explore the structural characteristics of films induced by DAS content, the films were detected using XRD technique, and the results were depicted in Fig. 4d. The diffraction patterns exhibited similar profiles, with two prominent crystalline peaks observed at approximately 11° and 20°. The peak at 2θ = 20° showed an increase followed by a decrease as the DAS content increased, while the peak at 2θ = 11° remained nearly unchanged except for DAS25-g-KF and DAS45-g-KF films. Meanwhile, crystallinity calculations also revealed a trend consistent with the intensity changes of the peak at 2θ = 20°. These variations could be attributed to the DAS content, KF degradation and their cross-linking interactions. While KF exhibited only one peak at 2θ = 20° and DAS showed three peaks at 2θ = 15°, 18°, 23.3°, the characteristic peaks of DAS were absent in the DAS-g-KF films, and a new peak emerged at 2θ = 11° as shown in Fig. 4f. Hence, it could be considered that the peak at 2θ = 11° primarily originate from the cross-linking between DAS and KF, which contributed positively to the film's performance. These findings were consistent with the FT-IR results. Notably, the DAS25-g-KF film exhibited the highest crystallinity value (68.91 %). So, it could be speculated that KF and DAS maybe play distinct roles in the films as the DAS content varied, with their interaction reaching an optimum at 25 % DAS. This explained, in part, why DAS25-g-KF film displayed the optimal performance.

**Fig. 5:**
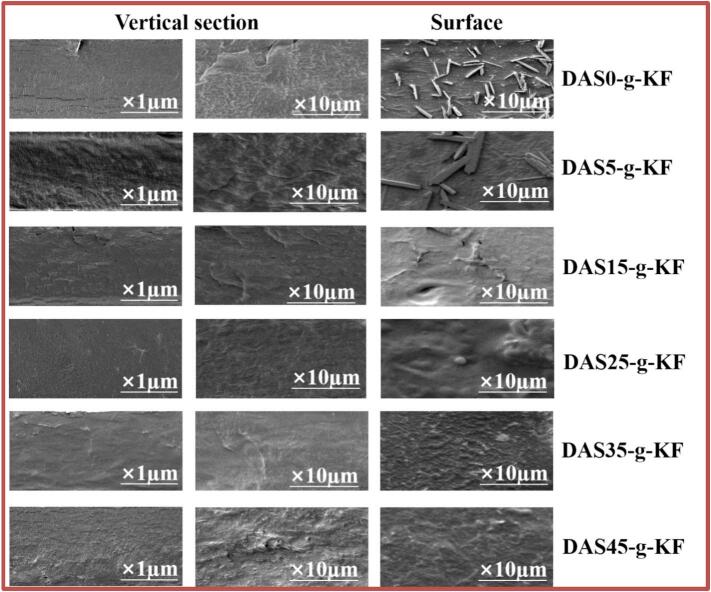
The SEM images of films including vertical section and surface.

The above speculation was further supported by scanning electron microscope (SEM) images of the films. As shown in Fig. 5, the vertical cross-section and surface of the films were examined. Overall, at a magnification of ×1 μm, the vertical cross-sections of all films exhibited relatively uniform structures, but the differences became apparent at a higher magnification (×10). For the DAS0-g-KF film, distinct cracks and a layered strucutrue were observed on the vertical cross-section, resulting from insufficient intermolecular cross-linking. However, as the DAS content increased, these cracks gradually diminished. Notably, when the DAS content reached 25 %, the structure became more compact and flat, indicating the formation of a denser and more effective cross-linked network between DAS and KF. However, when the DAS content exceeded 25 %, cracks reappeared, implying that the development of a multi-phase structure within the film and a shift in the intermolecular bonding pattern. These observations were consistent with the trends identified in the XRD and FT-IR test results. Similarly, the surface SEM images of the film also showed a comparable trend. Initially, numerous pine-needle-like free substances were visible, which gradually vanished upon the addition of DAS. The DAS25-g-KF film displayed the densest and most uniform surface morphology. Therefore, it could be concluded that the graft copolymer formed between DAS and KF played a crucial role in molecular bonding and arrangement during film formation, promoting a crystalline or crystal-like organization among the molecular chains and thereby endowing the film with superior comprehensive properties.

### 3.5 Thermal properties analysis

**Fig. 6:**
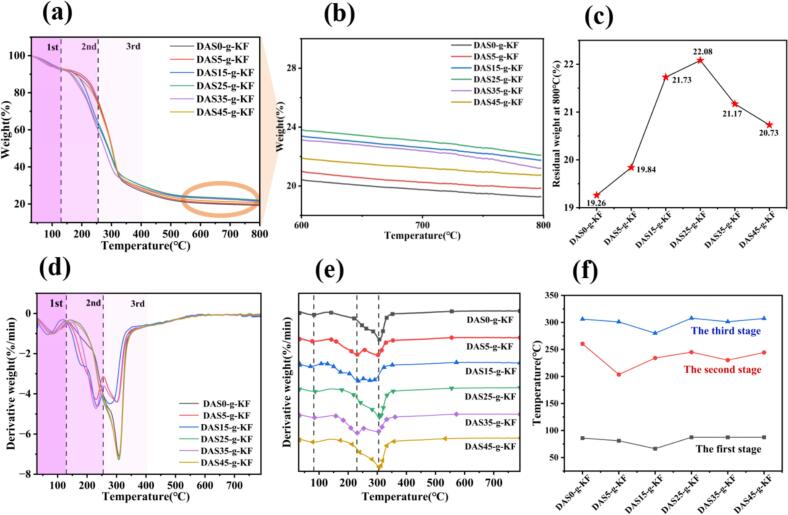
The TG and DTG curves of films. The TG curves of films (a); The magnified TG curves of films from 600 °C to 800 °C (b); The residual weight at 800 °C (c); The DTG curves of films (d); The three-stage decomposition temperature (e,f).

Thermal stability is a crucial parameter for evaluating the performance of composite films. In this work, thermogravimetry (TG) was employed to assess the thermal stability of the films. The thermal degradation process of the prepared films, as illustrated in Fig. 6a, could be divided into three main decomposition stages based on the thermal decomposition temperatures summarized in Fig. 6d. In general, the weight loss below 130 °C primarily resulted from the evaporation of water wrapped within the molecular structure of the films. As a result, no significant differences were observed in the peak temperature among all films. The weight loss in the temperature range of 200 °C ∼ 260 °C was associated with the thermal decomposition of glycerol and certain free groups or side-chain structure (Luo et al., 2025). The stage above 260 °C was regarded as the third region responsible for the film weight loss, which could be attributed to the thermal decomposition of the DAS grafted KF polymer.

As depicted in Fig. 6b and c, the residual weight of films was closely related to the DAS content. Although the addition of DAS significantly increased the residual weight, the extent of improvement varied due to difference in cross-linking. Consistent with other properties, the DAS25-g-KF film exhibited the highest residual weight, indicating more complete and sufficient cross-linking between DAS and KF. However, according to the results shown in Fig. 6e and f, the thermal degradation temperatures were similar, except for the DAS15-g-KF film, which showed a relatively lower peak temperature. It was worth noting that only two major decomposition peaks were observed in DAS0-g-KF, DAS25-g-KF and DAS45-g-KF films, with no distinct peak corresponding to glycerol decomposition as compared to the other films. This suggested that glycerol reacted more completely with the film at these specific ratios, further indicating that DAS grafted with KF at an appropriate proportion resulted in a more homogeneous microstructure. Moreover, the residual weight of the films remained nearly stable above 400 °C, and the fastest degradation region was between 150 °C and 350 °C, indicating that the prepared films in this study possessed good thermal stability.

### 3.6 Degradation analysis

**Fig. 7:**
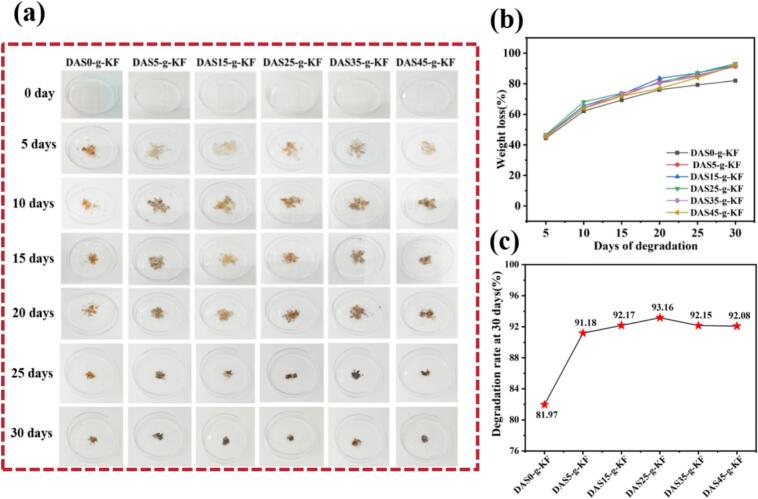
Degradation of films. Changes in film appearance (a); The weight loss of films (b); The degradation rate at 30 days (c).

As an important indicator, the degradability of films was closely related to their impact on the natural environment. So, in this work, the biodegradability of the films was investigated through a soil-burial experiment. The films were buried in soil, and their residual weight was measured at 5-day intervals over a period of 30 days. Fig. 7 showed the visual appearance and weight changes of the films during degradation. As indicated in Fig. 7a, the films were initially transparent but gradually acquired a coloration from the soil during the degradation progression. Meanwhile, the overall mass of the tested films steadily declined, as shown in Fig. 7b and c. This implied that the films exhibited high compatibility with the soil environment, leading to a continuous reduction in weight. Based on the degradation rate results, it was evident that the extent of degradation increased for all film samples with extended testing time. And films containing DAS exhibited a more significant increase in degradation rate compared to pure KF films, suggesting that DAS could promote the film degradation. In particular, the DAS25-g-KF film exhibited the highest degradation rate, reaching 93.16 % at 30 days. In conclusion, the addition of DAS would enhance the degradation performance of the films.

### 3.7 Preservation packaging application and oxygen permeability

**Fig. 8:**
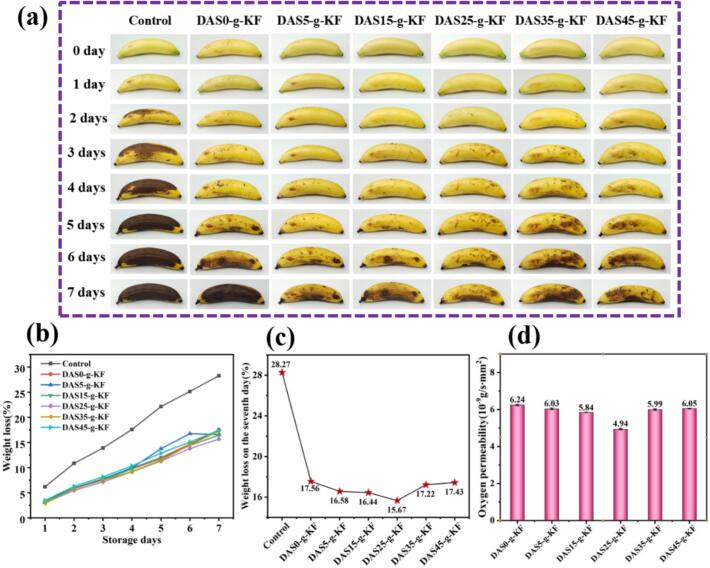
Films application in preserving bananas. Changes in appearance (a); The weight loss of tested bananas in 7 days (b); The weight loss rate of bananas at 7 days (c); The oxygen permeability of the films (d).

To assess the practical feasibility of the film for preservation packaging, bananas were selected as the tested subject and packaged using the prepared films. Fig. 8a presented the appearance of tested bananas over a 7-day storage period. In the control group, browning began on the second day, whereas bananas wrapped with the film retained their natural skin color after the same storage period. This browning phenomenon was primarily attributed to continuous respiration and water evaporation, which not only affects fruit quality but also reduces its economic value. (Liu et al., 2024). In addition to appearance, the weight changes of the bananas during the 7-day storage period were shown in Fig. 8b and c. The weight loss rate of the bananas gradually increased with extended storage time. In the control group, the weight loss rate reached 28.27 % after 7 days, while the values for bananas wrapped with DAS0-g-KF, DAS5-g-KF, DAS15-g-KF, DAS25-g-KF, DAS35-g-KF, and DAS45-g-KF film were 17.56 %, 16.58 %, 16.44 %, 15.67 %, 17.22 %, and 17.43 %, respectively. These results indicated that the addition of DAS significantly reduced the weight loss rate of bananas, particularly the DAS25-g-KF film had the lowest weight loss rate. This could be attributed to the high cross-linking density and improved water vapor barrier properties of the film. Moreover, the oxygen permeability of the film was also tested, the result was shown in Fig. 8d. The film demonstrated effective oxygen-barrier properties, which contributed positively to maintaining the freshness of the bananas. These findings indicated that DAS-g-KF films possessed excellent properties and showed significant potential for application in food packaging.

## 4 Conclusion

In this work, a series of bio-based films were developed using KF and DAS as the primary film-forming materials through constructing a redox system composed of ascorbic acid/hydrogen peroxide (AA/H_2_O_2_) with glycerol as a plasticizer. The tested results revealed that the films were colorless and transparent, and exhibited excellent overall physical and mechanical properties. Meanwhile, the content of KF and DAS significantly influenced the performance of films, with the 25 % DAS formulation showing the optimal performance. This was mainly attributed to the well-developed cross-linking structure between DAS and KF, as confirmed by FT-IR, XRD and SEM analyses. Furthermore, the TG results indicated that the films possessed excellent thermal stability, with the main thermal decomposition process starting at approximately 130 °C and reaching the maximum rate at around 300 °C. In addition, when these films were applied as packaging materials for banana preservation under ambient conditions, their performance was remarkable. This finding indicated their great potential for application in food packaging application. And the biodegradability of the films was also outstanding, achieving a degradation rate exceeding 90 % after 30 days. Therefore, the development of bio-based films using KF grafted DAS as the primary film-forming materials represented a highly promising approach, although the water resistance was still not outstanding. To address this defect, future research could explore the incorporation of hydrophobic substances, such as acrylic, silicon-based materials, PLA and paraffin wax, into the film matrix, or apply them as coatings on the surface of bio-based films.

## CRediT authorship contribution statement

**Xin Zhang:** Writing – original draft, Validation, Methodology, Data curation. **Jiawei Li:** Methodology, Investigation. **Hongyan Wang:** Visualization, Methodology, Investigation. **Xiaojian Zhou:** Methodology, Investigation, Funding acquisition, Conceptualization. **Liangjun Xiao:** Investigation, Data curation. **Hui Wang:** Writing – review & editing, Methodology, Conceptualization.

## Declaration of competing interest

The authors declare that they have no known competing financial interests or personal relationships that could have appeared to influence the work reported in this paper.

## Data Availability

All research data has been contained in files.
